# Thermodynamic Modeling of Gas Transport in Glassy Polymeric Membranes

**DOI:** 10.3390/membranes7030046

**Published:** 2017-08-19

**Authors:** Matteo Minelli, Giulio Cesare Sarti

**Affiliations:** Department of Civil, Chemical, Environmental and Materials Engineering (DICAM), Alma Mater Studiorum–University of Bologna, Via Terracini, 28-I-40131 Bologna, Italy; matteo.minelli@unibo.it

**Keywords:** gas solubility, gas permeability, thermodynamics, glassy polymers, NELF model

## Abstract

Solubility and permeability of gases in glassy polymers have been considered with the aim of illustrating the applicability of thermodynamically-based models for their description and prediction. The solubility isotherms are described by using the nonequilibrium lattice fluid (NELF) (model, already known to be appropriate for nonequilibrium glassy polymers, while the permeability isotherms are described through a general transport model in which diffusivity is the product of a purely kinetic factor, the mobility coefficient, and a thermodynamic factor. The latter is calculated from the NELF model and mobility is considered concentration-dependent through an exponential relationship containing two parameters only. The models are tested explicitly considering solubility and permeability data of various penetrants in three glassy polymers, PSf, PPh and 6FDA-6FpDA, selected as the reference for different behaviors. It is shown that the models are able to calculate the different behaviors observed, and in particular the permeability dependence on upstream pressure, both when it is decreasing as well as when it is increasing, with no need to invoke the onset of additional plasticization phenomena. The correlations found between polymer and penetrant properties with the two parameters of the mobility coefficient also lead to the predictive ability of the transport model.

## 1. Introduction

The analysis of the solubility and permeability of gases, vapors and liquids in polymeric phases is of remarkable relevance for various applications [1,2,3,4,5], and among the others for membrane-based gas separation [6]. The development of novel membrane materials, as well as the appropriate design of the separation process, requires a deep understanding of the solubility and diffusion mechanisms in the polymer phase. For this reason, modeling efforts are definitely required for the description of the experimental behaviors observed, in order to identify and possibly predict their correlation with relevant material and process parameters, such as temperature, pressure and gas composition.

The intrinsic nonequilibrium nature of glassy polymers requires dedicated modeling tools, as conventional approaches are not appropriate for a nonequilibrium phase. In spite of their need, very few models have been developed for the representation of gas solubility [7,8,9,10] and transport properties in glassy polymers [11,12,13,14]. Therefore, the Dual Mode Sorption model (DMS) [15,16] is still widely used mainly due to its very simple formulation, even if it relies on adjustable parameters that do not allow predictions of the trends observed.

More recently, an alternative approach for penetrant permeability and diffusivity in glassy polymers has been proposed, simply based on the fundamental transport equations [17]. The model considers the chemical potential gradient as the actual driving force for diffusive flux, so that the diffusion coefficient is taken as the product of a purely kinetic factor, the mobility coefficient *L*, and a thermodynamic factor, accounting for the concentration dependence of the penetrant chemical potential. Such a model has been developed in the framework of the nonequilibrium thermodynamics for glassy polymers (NET-GP) [18,19], which provides the required thermodynamic representation of the penetrant/polymer mixtures in nonequilibrium conditions.

The model has been already widely tested with various penetrants, namely, gases [20,21], vapors [22] and binary gas mixtures [23] on several glassy polymers, including conventional materials [20], blends and copolymers [24], high free volume glassy polymers and even semicristalline polymers [25]. Remarkably, the solid theoretical basis of the model allowed the derivation of a predictive tool for the a priori evaluation of the gas permeability in glassy membranes, based only on the correlations found between model parameters and the properties of pure penetrant and pure polymer [26].

Such a transport model represents a simple but effective tool for the description and prediction of gas permeability, and for the evaluation of its dependence on relevant process conditions as feed pressure, composition and temperature, required for the design and development of novel membrane materials, as well as for the optimization of gas separation processes.

In this work, the model has been described and applied in detail to the case of gas sorption and transport in three relevant glassy polymeric systems: (i) polysulfone (PSf), selected as a traditional and commercial membrane material; (ii) polyimide 2,2-bis(3,4-carboxyphenyl) hexafluoropropane dianhydride, 4,4-hexafluoro diamine (6FDA-6FpDA), and poly (phenolphthalein terephthalate) (PPh), selected as representative of innovative materials.

## 2. Theoretical Background

### 2.1. Solubility

The description of the thermodynamic behavior of polymer and penetrant mixtures in the glassy state can be provided by the NET-GP model, which offers a reliable and accurate method to evaluate the solubility of any low molecular weight species at any temperature, pressure and composition. The model suitably extends to the glassy state the applicability of an equation of state which isappropriate to describe the properties of equilibrium polymeric phases (e.g., polymer melts or rubbers). It is based on the proper use of the actual polymer density as a further state variable, required to account for the nonequilibrium behavior of polymeric glasses, in addition to the usual set of variables (i.e., *T*, *p*, and composition). It is worth pointing out that the NET-GP approach considers a uniform nonequilibrium metastable polymer phase, without any artificial differentiation among different populations of penetrant molecules, which are in fact all treated as dissolved in the polymer. Such noequilibrium approach has been widely employed as NELF model by using the lattice fluid equation of state (EoS) model by Sanchez and Lacombe [27,28], or as nonequilibrium perturbed hard sphere chain theory (NE-PHSC) [29] and nonequilibrium statistical associating fluid theory (NE-SAFT) [30] by using tangent spheres-based model perturbed hard sphere chain theory (PHSCT) [31] and statistical associating fluid theory (SAFT) [32,33], to predict the solubility behavior of gases [34], vapors [35], liquids [36] and gas mixtures [37,38] in all kinds of glassy polymers [39,40,41]. The general ability of the model to represent the observed behavior has been shown in all the cases inspected, even in the case of *S*-shape solubility isotherms sometimes shown by the sorption of alcohols in high free volume glasses [42,43]. Recently, the NELF model was also shown suitable to represent and predict sorption isotherms in glassy polymers at cryogenic temperatures [44].

In the practical cases considered in the present work, the NELF model will be used; the detailed equations of the model are summarized in Table 1 for the sake of brevity, and the pure polymer and pure penetrant model parameters are listed in Table 2 and Table 3.

For the NELF model pure polymers and pure penetrants are characterized by the pure component parameters (p_i_*, ρ_i_*, T_i_*) of the equilibrium Sanchez and Lacombe theory, and the mixture properties (p*, ρ*, T*) are obtained through the mixing rules used in the same model [27,28]. The pure component characteristic parameters are generally obtained by best fitting the equilibrium equation to pressure-volume-temperature (*pVT*) data above *T_g_* for the polymers, and to either *pVT* or vapor-liquid equilibrium data for the penetrant.

The density of the glassy polymer, $\rho_{2}^{NE}$, depends on the experimental conditions and on the history of the samples, as usual. For non-swelling penetrants, the density of the polymer phase at every pressure can be considered equal to the value of the pure unpenetrated polymer. In the case of swelling agents, as CO_2_ and hydrocarbons, the density of the polymer at every sorption pressure can be retrieved from parallel dilation experiments. In their absence, one may benefit from the experimental observation that the dependence of polymer density on penetrant pressure is commonly linear [45,46], so that a swelling coefficient, *k*_sw_, can be used to account for volume dilation in a simple and effective way (see Equation (1)):

(1) $$\frac{1}{\rho_{2}^{NE}(p_{1})}=\frac{\left(1-k_{\mathrm{sw}}p_{1}\right)}{\rho_{2}^{0}}$$

where $\rho_{2}^{0}\;$ is the density of the pure unpenetrated glassy polymer. In the absence of specific dilation data, the parameter *k*_sw_ can be adjusted virtually on one only solubility datum at high pressure, providing also an estimate of the swelling isotherm of the matrix at all other pressures, through Equation (1).

The swelling coefficient can also be also obtained in a completely predictive fashion by means of the model derived by Minelli and Doghieri [47].

Of course, the solubility isotherms of penetrant 1 is obtained from the phase equilibrium equation (Equation (2)):

(2) $$\mu_{1}^{NE(s)}\left(T,p,\omega_{1},\rho_{2}\right)=\mu_{1}^{Eq(g)}\left(T,p,y_{1}\right)$$

where $\mu_{1}^{NE\left(s\right)}$ is the nonequilibrium chemical potential of the penetrant 1 within the glass, in which the polymer density is ρ_2_, ω_1_ is the penetrant mass fraction, and $\mu_{1}^{Eq\left(g\right)}$ is its chemical potential in the gas phase whose mole fraction is *y_1_*.

### 2.2. Diffusivity

Under isothermal conditions the diffusive mass flux of penetrant 1, *J*_1_, in a binary mixture in polymer 2 is given by Equation (3):

(3) $$\;J_{1}=-\rho L_{12}\omega_{1}\nabla \left(\frac{\mu_{1}}{RT}\right)$$

where ρ is the density of the phase, μ_1_ is the chemical potential of penetrant 1 and *L*_12_ is its mobility coefficient, which is temperature and composition dependent; *R* and *T* are the universal gas constant and absolute temperature, respectively. By comparing Equation (2) with Fick’s law in Equation (4):

(4) $$\;J_{1}=-\rho D_{12}\nabla \omega_{1}$$

One has that the diffusion coefficient *D*_12_ is given by Equation (5):

(5) $$D_{12}=L_{12}\frac{\partial \mu_{1}/RT}{\partial \ln\omega_{1}}\equiv L_{12}\alpha_{12}$$

where α_12_ defined in Equation (5) is the thermodynamic factor.

In the case of a polymer glass there is no need to arbitrarily consider different types of penetrant molecules, since the phase is macroscopically continuous and homogeneous, just as it was in the case of the solubility calculations. One has to use the suitable expression for the chemical potential which holds for the polymeric phase under consideration, e.g., the NELF model. In addition, the proper dependence of the mobility coefficient on composition must be considered. All the cases inspected thus far [17,20,21,22,23,24,25,26] indicate that a simple exponential dependence of penetrant mobility coefficient on penetrant mass fraction is sufficient to describe the broad spectrum of observed behaviors, including both cases in which steady state permeability is either decreasing or increasing with upstream pressure. Therefore, even though a more general assumption might be considered, we will confine our attention to the simple case in which the exponential dependence reported in Equation (6) holds:

(6) $$L_{12}=L_{12}^{0}\times \exp\left(\beta_{12}{\;\omega }_{1}\right)$$

where $L_{12}^{0}$ and β_12_ are the pre-exponential factor and the plasticization parameter of penetrant 1 in polymer 2, respectively; they are the only two parameters used in the transport model. It is worth pointing out that the sensitivity factor of mobility versus penetrant mass fraction, $d\ln L_{12}/d\ln\omega_{1}$, representing the percent variation experienced by mobility for a unit percent variation of penetrant mass fraction, is given by the product $\beta_{12}\;\omega_{1}$ and not simply by the plasticization factor β_12_ alone. Accordingly, significant variations in the mobility coefficient are obtained for relatively high values of the product $\beta_{12}\;\omega_{1}$ , and not simply from high values of the plasticization factor β_12_ alone

Use of Equations (5) and (6) in Equation (4) leads to the explicit expression for the diffusive mass flux of penetrant 1 in the glassy phase under consideration, provided the nonequilibrium expression for the chemical potential is considered.

The steady state permeability, *P*_12_, of species 1 is often considered as a valuable property to represent the membrane performance:

(7) $$P_{12}=\frac{J_{1,ss}l}{M_{1}\;\left(p_{1}^{u}-p_{1}^{d}\right)}$$

In Equation (7), subscripts *u* and *d* label upstream and downstream properties, *P* is pressure and *l* is the membrane thickness; the molecular mass is introduced since commonly permeability is expressed on a molar basis. According to the transport model presented, the explicit expression for the penetrant permeability at steady state is easily obtained as follows:

(8) $$P_{12}=\frac{1}{M_{1}\;\left(p_{1}^{u}-P_{1}^{d}\right)}\int_{p_{1}^{d}}^{p_{1}^{u}}\rho_{2}\;L_{12}^{0}\exp\left(\beta_{12}{\;\omega }_{1}\right)\;\frac{\omega_{1}}{p_{1}}\;z_{1}\;dp_{1}$$

where *z*_1_ is the compressibility factor of species 1 in the gas phase. Comparison between experimental permeability data and calculations made by using Equation (8) allows the evaluation of the two parameters of the transport model.

### 2.3. Correlations

The present model is derived on a fundamental theoretical basis, making use of meaningful and physically sound parameters. It has been shown, indeed, that both $L_{12}^{0}$ and β_12_ are correlated to the properties of pure penetrant and pure polymer [26].

In more detail, the mobility coefficient of each penetrating gas (e.g., CO_2_) depends on the fractional free volume (FFV) of the polymer matrix, which can be evaluated by means of Bondi’s method [52]:

(9) $$L_{0,{\mathrm{CO}}_{2}}=A_{{\mathrm{CO}}_{2}}\times \exp\left(\frac{B_{{\mathrm{CO}}_{2}}}{FFV}\right)$$

On the other hand, the mobility of various gases in the same polymer matrix typically scales with the molecular size of the penetrant species, following a power law dependence with respect to the penetrant molar critical volume *V_c_* [53]. A simple relationship was thus derived [26], considering CO_2_ as reference penetrant:

(10) $$L_{i2}^{0}=L_{{\mathrm{CO}}_{2}}^{0}{\left(\frac{V_{c,{\mathrm{CO}}_{2}}}{V_{c,i}}\right)}^{\eta }$$

in which η is the sieving ability of the matrix, and represents a polymer dependent property. Its value, indeed, correlates well with the characteristic temperature $T_{2}^{*}$ of the pure polymer species [26] following the expression provided in Equation (11):

(11) $$\eta =\eta_{0}\times \exp\left(\frac{T_{2}^{*}}{T}\right)$$

$T_{2}^{*}$ is, by definition, proportional to the polymer characteristic energy $\varepsilon_{2}^{*}$ that represents the energy required to create a vacancy in the polymer lattice [27].

Therefore, Equations (9)–(11) provide a simple tool for the prediction of the mobility coefficient in the limit of infinite dilution, based only on the pure polymer and penetrant characteristics, often readily available and measured in independent experimental tests.

In a previous work, a large portion of the experimental transport data available in the literature has been analyzed, and the values of the model constants introduced above, $A_{{\mathrm{CO}}_{2}}$, $B_{{\mathrm{CO}}_{2}}$, η_0_, have been determined [26]. Hence, the permeability of a generic penetrant/polymer couple can be evaluated predictively in the limit of low upstream pressure:

(12) $$P_{12}^{0}=\frac{\rho_{2}^{0}\;}{M_{1}}\;S_{1}^{0}\;L_{{\mathrm{CO}}_{2}}^{0}\;{\left(\frac{V_{c,{\mathrm{CO}}_{2}}^{}}{V_{c,1}}\right)}^{\eta_{0}\times \exp\left(T_{2}^{*}/T\right)}$$

## 3. Results

The solubility and permeability of various gases in glassy polymeric membranes are analyzed and described hereafter by means of the NELF model and of the transport model, respectively. CO_2_, N_2_ and CH_4_ solubility and permeability in PSf, PPh and 6FDA-6FpDA are considered first, comparing experimental and modeling results for the different glassy polymers examined; then, other penetrants, either light gases (e.g., He, H_2_, Ar, O_2_) or higher hydrocarbons (C_2_H_4_, C_2_H_6_, C_3_H_6_ and C_3_H_8_) are analyzed. Solubility and permeability data were obtained from published works [54,55,56,57,58,59].

### 3.1. CO_2_, CH_4_ and N_2_ Solubility and Permeability

**Figure 1:**
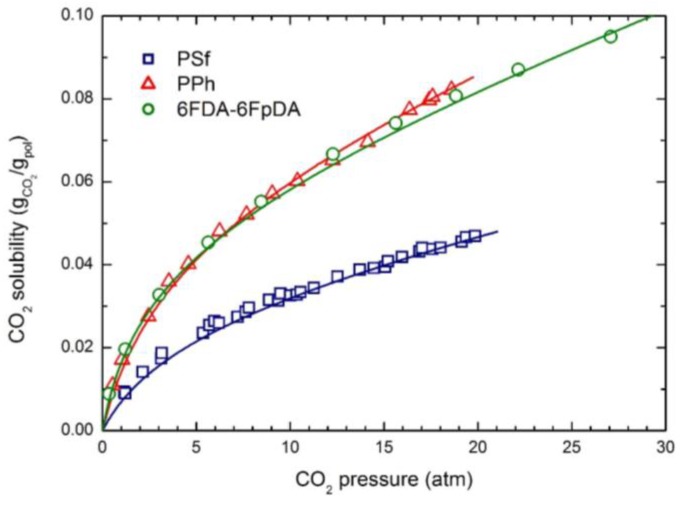
CO_2_ solubility in the three glassy polymer membranes at 35 °C: Experimental data and NELF model curves.

**Figure 2:**
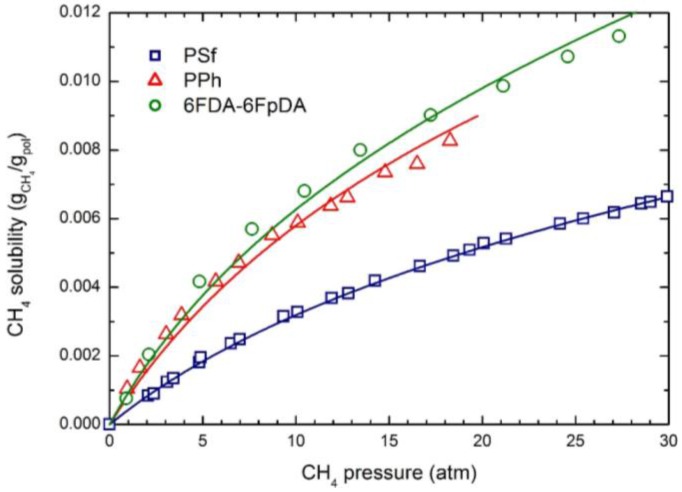
CH_4_ solubility in the three glassy polymer membranes at 35 °C: Experimental data and NELF model curves.

**Figure 3:**
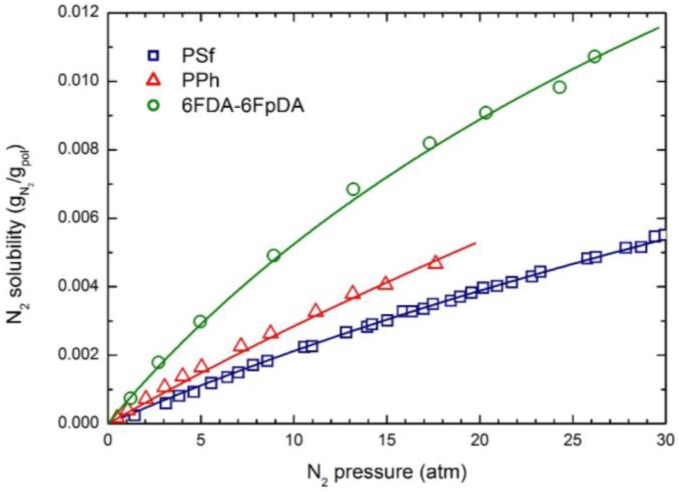
N_2_ solubility in the three glassy polymer membranes at 35 °C: Experimental data and NELF model curves.

The experimental CO_2_ solubility at 35 °C in the three glassy polymers considered is reported in Figure 1; the curves were obtained by the NELF model calculations with the pure component parameters reported in Table 2, and the binary interaction parameters *k*_1,2_ and swelling coefficients *k*_sw_ reported in Table 4. CH_4_ and N_2_ solubility at 35 °C in the three polymers are reported in Figure 2 and Figure 3, together with the NELF model curves. As one can see, the NELF model provides an excellent description of the experimental behavior for all penetrant/polymer couples inspected.

The appreciable difference observed between CO_2_ solubility in PSf and in the other two polymers is attributed mainly to the difference in the fractional free volume (FFV) of the three glassy matrices, being that of PSf quite lower than that of the others two. Interestingly, such an effect is even clearer in the case of light gases, as the penetrant uptake scales very well with the polymer FFV, so that the CH_4_ and N_2_ solubilities are appreciably larger in 6FDA-6FpDA than in PPh, and those in PSf are the lowest.

Remarkably, CO_2_ is able to induce a significant dilation of all polymer matrices, as indicated by the rather large swelling coefficients calculated by the NELF model from the experimental solubility data (Table 4), while much lower values are obtained for CH_4_, and almost no swelling is associated with N_2_ sorption.

The analysis of CO_2_, CH_4_ and N_2_ solubility in the three glassy polymers selected leads to the determination of the model parameters required for the description of the chemical potential of each penetrant in the glassy phases, which is also required to calculate the thermodynamic factors used in the transport model.

**Figure 4:**
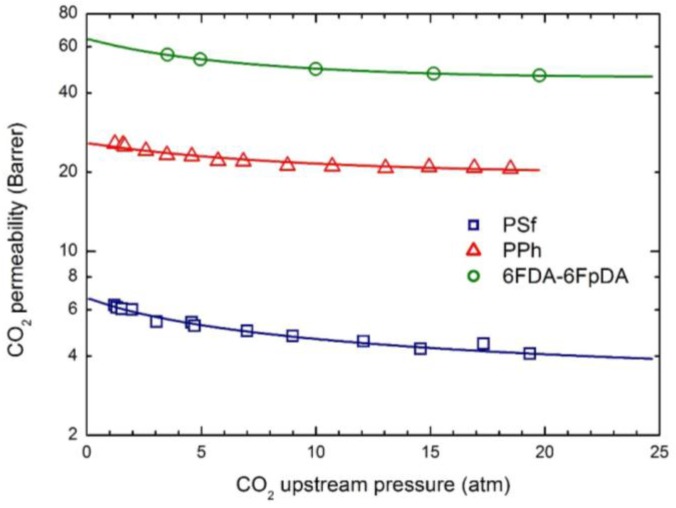
CO_2_ permeability in the three glassy polymer membranes at 35 °C: Experimental data and transport model curves (Equation (8)).

**Figure 5:**
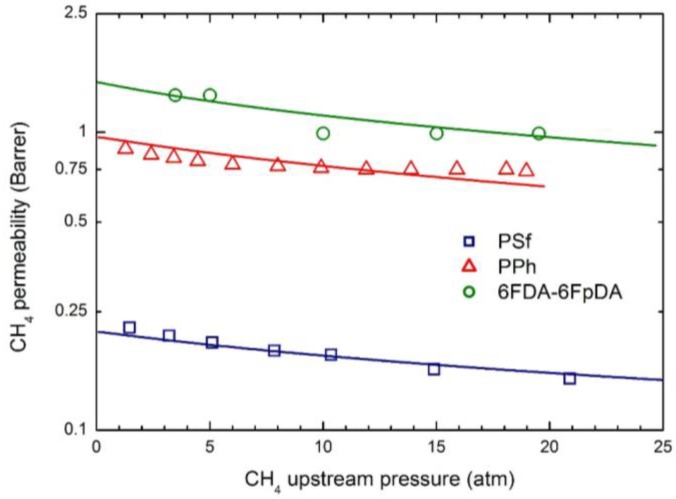
CH_4_ permeability in the three glassy polymer membranes at 35 °C: Experimental data and transport model curves (Equation (8)).

**Figure 6:**
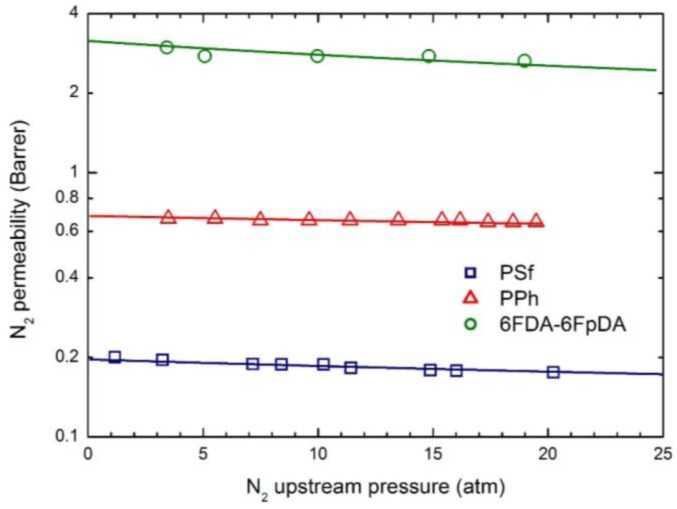
N_2_ permeability in the three glassy polymer membranes at 35 °C: Experimental data and transport model curves (Equation (8)).

The permeability dependence on upstream pressure for CO_2_, CH_4_ and N_2_ in the membranes selected is reported in Figure 4, Figure 5 and Figure 6, together with the curves obtained by the transport model presented above. In all cases, the two parameters $L_{12}^{0}$ and β_12_ have been obtained by best fitting the model results to the experimental data; the values obtained are listed in Table 5.

Remarkably, the model describes the experimental behavior very accurately using only two parameters, the infinite dilution mobility coefficient, $L_{12}^{0}$, and the plasticization factor β_12_.

As often observed experimentally, and as already discussed in previous works [17], gas permeability can present any kind of dependence on penetrant upstream pressure: it may be practically constant, as well as either a decreasing or an increasing function, and even may show a non-monotonous behavior going through a minimum point (the so-called “plasticization” point). Our transport model is able to describe all the different possible behaviors; according to it the decreasing trend is dominated by the solubility coefficient behavior, the increasing trend is dominated by the mobility behavior, while the non-monotonous dependence on upstream pressure is associated with a transition from an initial solubility dominated behavior to a subsequent mobility controlled behavior. Here, CO_2_ permeability shows a marked decreasing function with the penetrant upstream pressure, while for N_2_, practically constant trends are registered; intermediate behaviors are observed for CH_4_. Such features are all well described by the transport model. Quite large values of the plasticization factor β_12_ are calculated for the most soluble penetrant, CO_2_, although the sensitivity factor for mobility, $\beta_{12}\;\omega_{1}$, is never high enough and the behavior remains solubility driven; on the other hand much lower values of β_12_ are obtained for CH_4_ and N_2_.

The mobility is a purely kinetic property, so that its value in the limit of infinite dilution is related to the penetrant dimensions (often measured by penetrant critical volume *V_c_*) and the polymer excess of free volume (described by FFV). Therefore, *L*_0_ values of CO_2_ is appreciably larger than those of CH_4_ in all glassy systems inspected, while, on the other hand, the lager the FFV, the larger the mobility coefficient, for all gases.

Qualitatively, the plasticization of the glassy polymer at high penetrant pressure is related to the swelling induced in the matrix; consequently, β_12_ values are significantly larger for the more condensable and soluble penetrants (CO_2_ > CH_4_ > N_2_), and they typically scale with the swelling coefficients calculated from the analysis of gas sorption behavior.

### 3.2. Solubility and Permeability of Other Gases in PSf

The sorption and transport behavior of other gases have also been retrieved from the literature, and analyzed by means of the appropriate models recalled above, as a further test of the procedure presented.

**Figure 7:**
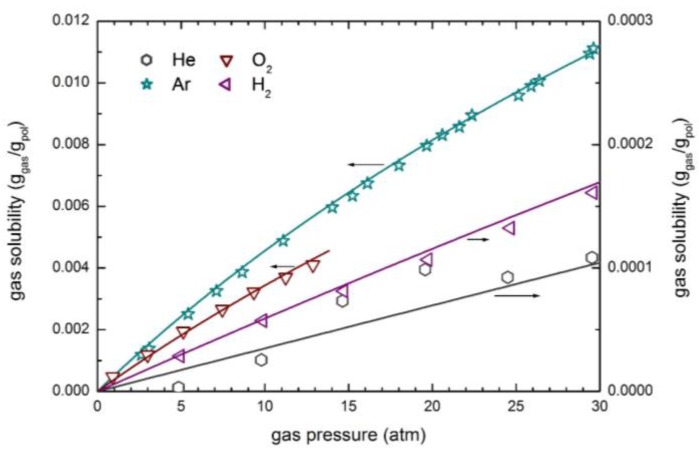
Gas solubility in PSf at 35 °C: Experimental data and NELF model curves.

The solubility and permeability of light gases (He, H_2_, O_2_ and Ar) have been considered in detail. The experimental solubility data from various sources [54,55,60] have been analyzed by the NELF model. Figure 7 illustrates the very good accuracy of the NELF model in the description of gas solubility isotherms, which are practically linear for almost all penetrants, with only a slight downturn observed for Ar at the higher pressures. As expected, these gases are not able to produce any swelling in the PSf; correspondingly null or very small values of the swelling coefficient *k*_sw_ are obtained (Table 4).

**Figure 8:**
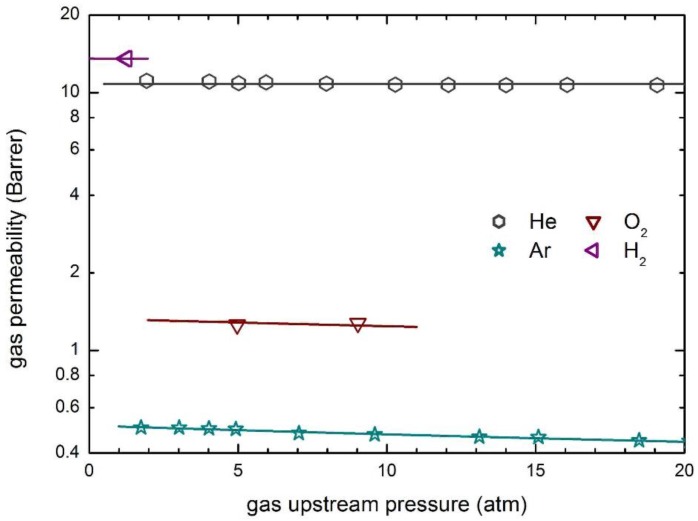
Gas permeability in PSf at 35 °C: Experimental data and transport model curves (Equation (8)).

The transport behavior of the same penetrants in glassy PSf is then reported in Figure 8, which also includes the curves obtained by model calculations. The observed permeabilities are practically constant at the different upstream pressures considered and only a slightly decreasing behavior is obtained for the penetrant Ar.

Zero value of the plasticization factors of the polymer matrix have been obtained (Table 5), and constant values of the mobility coefficient are able to describe well the whole experimental data in the pressure range inspected. Furthermore, as otherwise expected, the mobility coefficients of He and H_2_ are significantly larger than those of the other penetrants, as a consequence of their much smaller molecular dimensions.

### 3.3. Solubility and Permeability of Other Gases in PPh

**Figure 9:**
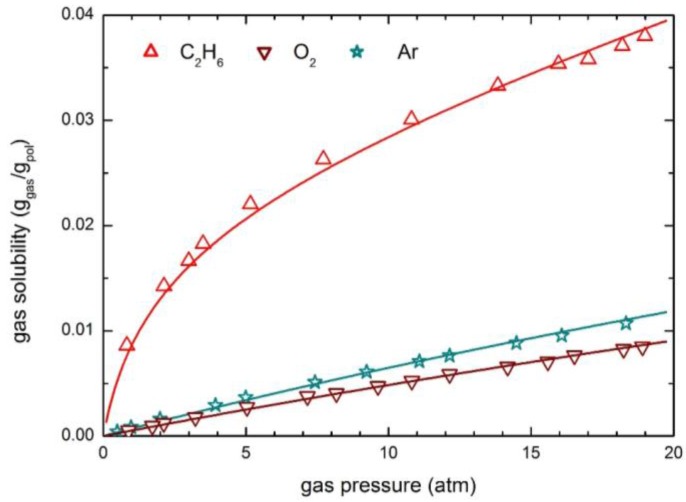
Gas solubility in PPh at 35 °C: Experimental data and NELF model curves.

The solubility and permeability behaviors of O_2_, Ar and C_2_H_6_ in glassy PPh at 35 °C are also analyzed. The experimental sorption isotherms are compared with the curves obtained by the NELF model in Figure 9. Also in this case the solubility behavior of light gases (O_2_ and Ar) is practically linear, while a significant downward concavity is observed for the more condensable species C_2_H_6_.

Notably, ethane solubility in PPh is appreciably larger than that of O_2_ and Ar, and the sorption process produces a significant dilation of the polymer matrix, as the swelling coefficient *k*_sw_ is comparable to that of CO_2_ (Table 4), so that an appreciable mobility dependence on concentration is expected, i.e., a quite large plasticization factor β_12_.

**Figure 10:**
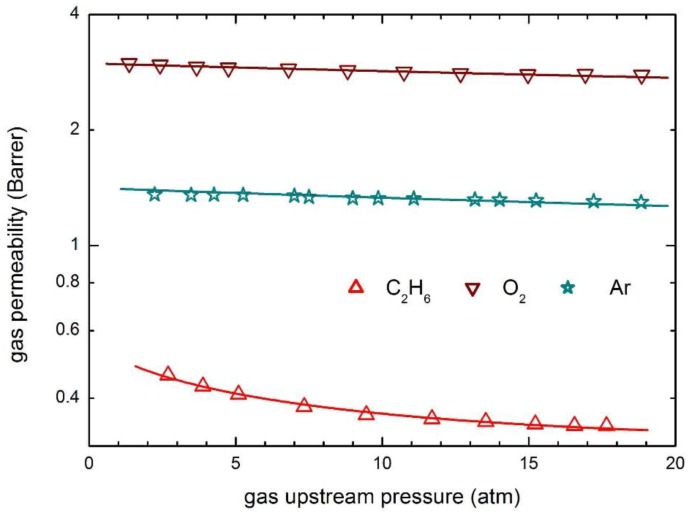
Gas permeability in poly (phenolphthalein terephthalate) (PPh) at 35 °C: Experimental data and transport model curves (Equation (8)).

Figure 10 illustrates the permeability isotherms of O_2_, Ar and C_2_H_6_ in PPh, comparing the experimental data with model calculations. Interestingly, all three permeability curves are decreasing functions of the penetrant upstream pressure, but, while those of O_2_ and Ar are practically linear, that of C_2_H_6_ shows a convex behavior, very similar to that of CO_2_ (Figure 4).

Clearly, the transport model describes well the experimental permeability behavior of Ar and O_2_ with a constant mobility coefficient, i.e., zero value of the plasticization factor is observed in the pressure range inspected, while a quite large β_12_ is obtained for ethane.

### 3.4. Solubility and Permeability of Other Gases in 6FDA-6FpDA

**Figure 11:**
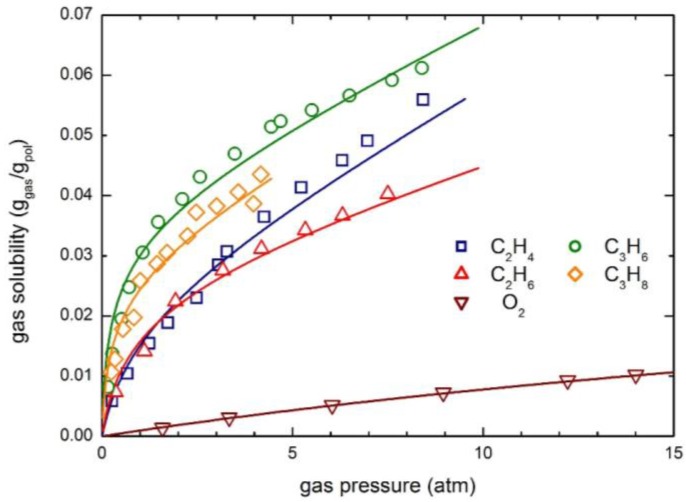
Gas solubility in 6FDA-6FpDA polyimide at 35 °C: Experimental data and NELF model curves.

The experimental solubility data of O_2_, C_2_H_4_, C_2_H_6_, C_3_H_6_ and C_3_H_8_ have been retrieved from the literature and analyzed by means of the NELF model; the results obtained are illustrated in Figure 11. The O_2_ solubility isotherm is quite linear and the overall uptake is rather limited (up to 1 wt % at 15 atm) in the pressure range investigated; on the contrary, the hydrocarbon solubility curves show a concave behavior towards the pressure axis, typical of glassy polymers and very similar to that of CO_2_ (Figure 1). Interestingly, propane and propylene are appreciably more soluble than ethane end ethylene, and the expected swelling, as calculated by the NELF model, is also much larger.

**Figure 12:**
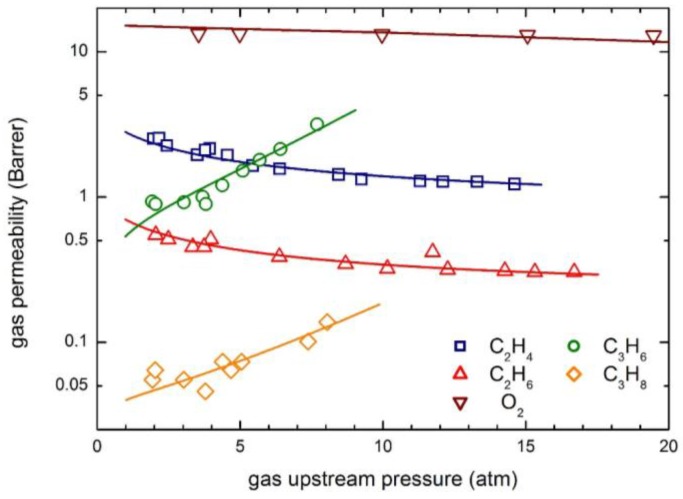
gas permeability in 6FDA-6FpDA polyimide: Experimental data and transport model curves (Equation (8)).

The permeability behaviors of the same components are reported in Figure 12, which contains the experimental data and the curves obtained from the transport model calculations. Remarkably, very different trends are observed for the various penetrants, since with increasing upstream pressure, permeability is practically constant for O_2_, is a decreasing function for C_2_H_4_ and C_2_H_6_ and is an increasing function for C_3_H_6_ and C_3_H_8_. All the different types of behavior are well described by the transport model considered, by means of two model parameters $L_{12}^{0}$ and β_12_ only, with no need to arbitrarily invoke an additional fictitious phenomenon (the so-called “plasticization”) as it is required by using the DMS model [15,16].

As often observed, relatively condensable gases such as hydrocarbons, largely soluble in the polymer matrices, induce significant swelling of the membranes, so that the mobility (and the diffusion) coefficients are strong increasing functions of penetrant concentration. Such an effect can be qualitatively inferred by the analysis of the permeability behavior, which shows an increasing trend for two heavier hydrocarbons. Indeed, quite large plasticization factors β_12_ are obtained for hydrocarbons (Table 5), especially for C_3_H_6_ and C_3_H_8_, which exhibit β_12_ values in excess to 110, in line with the relatively large solubility of such penetrants and with their strong swelling ability (see Table 4). The mobility coefficient at infinite dilution, on the other hand, is significantly lower for hydrocarbon with respect to lighter penetrants, due to their significantly larger molecular dimensions.

### 3.5. General Correlations

As already discussed, the model parameters $L_{12}^{0}$ and β_12_ are not simply fitting coefficients in a mathematical expression but have actually a real physical meaning since they are strongly and consistently correlated to the properties of both pure penetrant and pure polymer [26].

**Figure 13:**
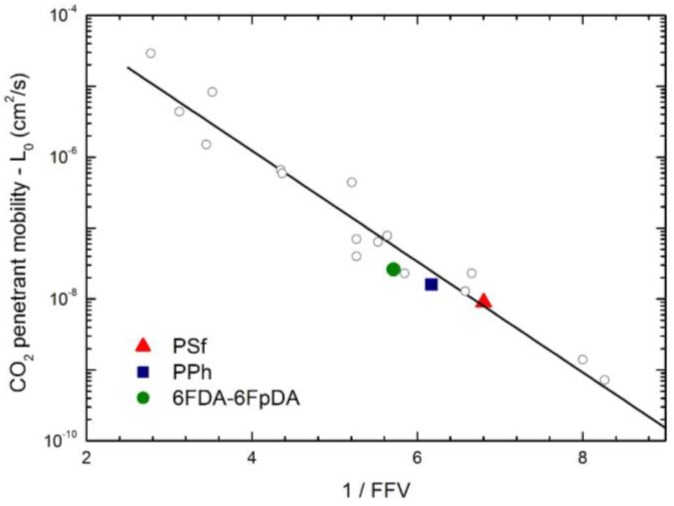
Correlation of CO_2_ infinite dilution mobility coefficient *L*_0_ at 35 °C with polymer fractional free volume (FFV) (Equation (9)): Solid points are for the polymers analyzed in this work, and open diamonds are from ref. [26].

The infinite dilution penetrant mobility is correlated with the availability of the excess of free volume in the matrix, a relevant property of glassy polymers, and, for any given penetrant, the larger the fractional free volume FFV, the higher its infinite dilution mobility coefficient. Figure 13 reports the general correlation which was found for the infinite dilution mobility coefficient of CO_2_ versus the FFV of the glassy matrix, showing that the values of the three glassy polymers investigated in this work follow the correlation rather well [24,26].

**Figure 14:**
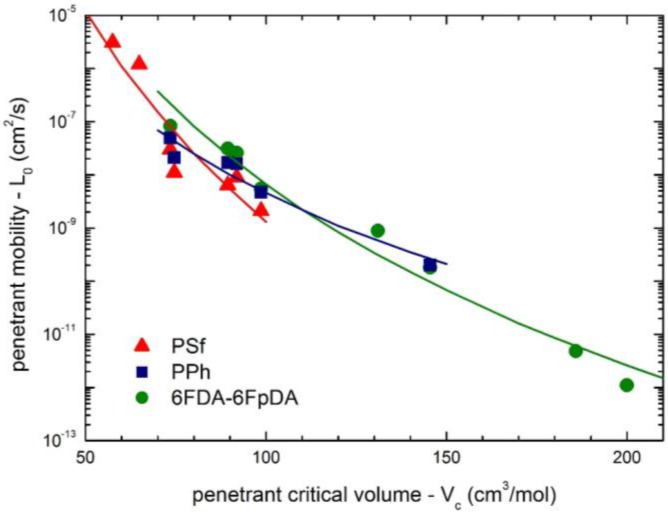
Correlation of penetrant infinite dilution mobility coefficient *L*_0_ at 35 °C with penetrant molar volume at the critical point (*V*_c_) for the three polymer systems inspected (Equation (10)).

Furthermore, it has been already discussed that the size of the penetrant also affects considerably its mobility coefficient, and the larger the penetrant, the lower is the value of *L*_0_. Figure 14 reports the correlation between penetrant molar volume at the critical point and the mobility coefficient at infinite dilution for all the penetrant/polymer couples analyzed in this work. As one can see, the *L*_0_ data follow a power law dependence versus penetrant *V*_c_ for each polymer system (Equation (10)), leading thus to sieving factors η equal to 13.2, 7.6 and 11.3 for PSf, PPH and 6FDA-6FpDA, respectively.

Clearly, the correlations considered appear physically sound although not always very accurate, and some deviations can be observed either in Figure 13 or Figure 14; that is mainly associated with the fact that (i) the method for the estimation of the FFV is quite approximated; and (ii) the representation of molecular size by its molar critical volume alone is somewhat crude; moreover, no interaction among penetrant molecule and membrane macromolecules have been considered yet. However, the trends observed are rather clear and consistent and can provide some useful information based only on few properties of the penetrant (i.e., critical volume) and of the polymer (i.e., density and molecular structure) in a purely predictive fashion.

### 3.6. Model Prediction of Gas Permeability

The present model approach is able to evaluate the gas permeability at low pressure with a very simple and purely predictive method, exploiting the correlations previously derived [26] and described above. Therefore, only on the basis of pure polymer and penetrant properties, the model provides *a priori* estimate of the gas permeability by means of Equation (12), with no need of any sorption or transport measurements. Such a procedure has been validated for CO_2_, CH_4_ and N_2_ on a quite large number of glassy polymers [26].

**Figure 15:**
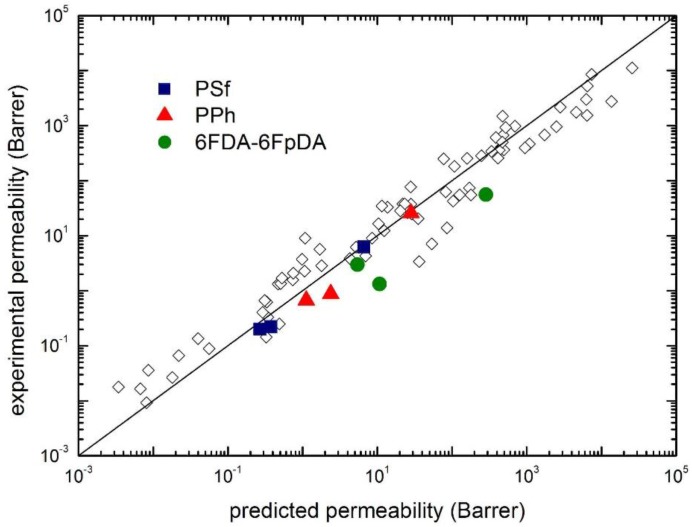
Comparison of experimental CO_2_, CH_4_ and N_2_ permeability (at 1 atm upstream pressure and 35 °C) with the values predicted by the transport model (Equation (12)): Solid points are for the polymers analyzed in this work, and open diamonds are from ref. [26].

The application of such method to the cases inspected in this work is illustrated in Figure 15, which reports a parity plot from the experimental and the predicted gas permeability in PSf, PPh and 6FDA-6FpDA, as well as in various glassy polymers. In the model predictions reported in Figure 15, calculations were made by considering the binary interaction parameter *k*_12_ given by its first order approximation (*k*_12_ = 0), as a pure blind prediction, instead of using its actual value obtained from the solubility isotherm.

As one can see, the model provides a reasonably good estimation of the experimental permeability, especially for PSF and PPh, while poorer results are obtained for 6FDA-6FpDA. It is worthwhile to recall, however, that this is the most simplified approach for the evaluation of gas permeability, in order to be fully predictive. Some improvements are indeed possible in order to increase the accuracy of the estimation, such as the independent evaluation of the binary interaction coefficient *k*_12_ (here considered 0), or the evaluation of one permeability value of, e.g., CO_2_.

## 4. Conclusions

The solubility and permeability of different penetrants, including light gases, CO_2_ and hydrocarbons, have been considered in three glassy polymers of interest for membrane gas separations, PSf, PPh and 6FDA-6FpDA. The solubility isotherms were accurately described by the NELF model in all cases including the presence of significant solute-induced swelling.

The permeability isotherms were calculated through a fundamental general model for binary mixtures, requiring that diffusivity is the product of a thermodynamic factor and a mobility coefficient. The same NELF model was provided for the calculation of the thermodynamic factor, while the mobility coefficient is considered concentration-dependent through a simple exponential relationship, containing two parameters only. The model is able to represent well all the behaviors observed, both when permeability is a decreasing function of upstream pressure as well as when it is an increasing function. The two parameters of the transport model follow general correlations with the properties of pure polymer and pure penetrant, which allow a predictive use of it.

Noticeably, in all cases both for solubility and for permeability, the glassy polymeric phase is considered macroscopically homogeneous with no need to artificially differentiate among different populations of penetrant molecules.

## Figures and Tables

**Table 1:** Definition of the different properties as well as main relationships for the Sanchez-Lacombe/NELF model.

Type of Phase	Symbol	Name	Definition/Property
Pure component *i*	ρ_i_*, p_i_*, T_i_*	characteristic density, pressure and temperature of pure component *i*	—
	*r_i_^0^*	number of lattice sites occupied by a mole of pure component *i*	$r_{i}^{0}=\frac{M_{i}}{\rho_{i}^{*}v_{i}^{*}}$
	v_i_*	volume occupied by a mole of lattice sites of pure substance	$v_{i}^{*}=\frac{RT_{i}^{*}}{p_{i}^{*}}$
	ω*_i_*	mass fraction of *i*	—
	φ*_i_*	volume fraction of *i*	$\phi_{i}=\frac{\omega_{i}/\rho_{i}^{*}}{\sum_{i}\omega_{i}/\rho_{i}^{*}}$
Multicomponent mixtures	ρ*	characteristic density of the mixture	$\frac{1}{\rho^{*}}=\frac{\omega_{1}}{\rho_{1}^{*}}+\frac{\omega_{2}}{\rho_{2}^{*}}$
	p*	characteristic pressure of the mixture	$p^{*}=\sum_{i}\varphi_{i}p_{i}^{*}-\frac{1}{2}\sum_{i}\varphi_{i}\sum_{j\neq i}\varphi_{j}\times \Delta p_{ij}^{*}$
	$\Delta p_{ij}^{*}$	binary parameter	$\Delta p_{12}^{*}=p_{1}^{*}+p_{2}^{*}-2\left(1-k_{12}\right)\sqrt{p_{1}^{*}\times p_{2}^{*}}$
	*r*	molar average number of lattice sites occupied by a molecule in the mixture	$r=\sum_{i}x_{i}r_{i}$
	T*	characteristic temperature of the mixture	$T^{*}=\frac{p^{*}}{r}\sum_{i}x_{i}r_{i}^{0}\frac{T_{i}^{*}}{p_{i}^{*}}=\frac{p^{*}v^{*}}{R}$
	v*	average close-packed mer molar volume in the mixture	$v^{*}=\frac{RT^{*}}{p^{*}}$
	$A^{Eq}$	total Equation elmholtz free energy	$\frac{A^{Eq}}{rNRT^{*}}=-\tilde{\rho }+T\left[\left(\frac{1}{\tilde{\rho }}-1\right)\ln\left(1-\tilde{\rho }\right)+\frac{1}{r}\ln\left(\tilde{\rho }\right)+\frac{\phi_{1}}{r_{1}}\ln\left(\phi_{1}\right)+\frac{\phi_{2}}{r_{2}}\ln\left(\phi_{2}\right)\right]$
	$\mu_{i}^{NE}$	chemical potential of penetrant 1 in the non Equation glass 2	$\frac{\mu_{1}^{NE}}{RT}=\ln\left(\tilde{\rho }\phi_{1}\right)-\ln\left(1-\tilde{\rho }\right)\left[r_{1}^{0}+\frac{r_{1}-r_{1}^{0}}{\tilde{\rho }}\right]-r_{1}-\tilde{\rho }\frac{r_{1}v_{1}^{*}}{RT}\left[p_{1}^{*}+p_{2}^{*}-\phi_{2}\Delta p_{1,2}^{*}\right]$

**Table 2:** Penetrant and polymer characteristic parameters of the Sanchez Lacombe lattice fluid EoS/NELF model.

Penetrant/Polymer	T* (K)	p* (MPa)	ρ* (g/cm^3^)	Ref.
PSf	820	560	1.318	[18]
PPh	650	550	1.470	[26]
6FDA-6FpDA	785	720	1.683	[21]
CO_2_	300	630	1.515	[18]
CH_4_	215	250	0.500	[48]
N_2_	145	160	0.943	[49]
He	9.3	4.0	0.148	[50]
H_2_	4.6	37	0.078	[48]
O_2_	170	280	1.290	[51]
Ar	190	180	1.400	[50]
C_2_H_4_	295	345	0.68	[49]
C_2_H_6_	320	330	0.640	[51]
C_3_H_6_	345	379	0.755	[28]
C_3_H_8_	375	320	0.690	[51]

**Table 3:** Physical and thermodynamic properties of penetrants and polymer species considered in this work.

Polymer	*T_g_* (K)	FFV	ρ^2^ (g/cm^3^)	Penetrant	*T_c_* (K)	*V_c_* (cm^3^/mol)
PSf	185	0.147	1.235	CO_2_	304.2	91.9
PPh	299	0.162	1.291	CH_4_	190.6	98.6
6FDA-6FpDA	287	0.175	1.485	N_2_	126.2	89.4
–	–	–	–	He	5.19	57.5
–	–	–	–	H_2_	33.18	64.9
–	–	–	–	O_2_	154.6	73.5
–	–	–	–	Ar	150.8	74.6
–	–	–	–	C_2_H_4_	282.5	131.0
–	–	–	–	C_2_H_6_	305.3	147.0
–	–	–	–	C_3_H_6_	365.2	185.9
–	–	–	–	C_3_H_8_	369.9	200.0

**Table 4:** Penetrant/polymer binary interaction parameters and swelling coefficients at 35 °C for the lattice fluid/NELF EoS models.

Polymer	Penetrant	*k*_12_	*k*_sw,12_ × 10^4^ (atm^−1^)	Ref. Exp.
PSf	CO_2_	0.013	9.5	[54]
	CH_4_	0.015	1.1	
	N_2_	−0.020	0.27	
	Ar	0.045	0.53	
	He	−0.900	0.0	[60]
	H_2_	−0.400	0.0	
	O_2_	0.025	0.35	[56]
PPh	CO_2_	−0.020	19	[57]
	CH_4_	−0.005	2.3	
	N_2_	0.012	1.1	
	Ar	0.065	1.9	
	O_2_	0.050	2.4	
	C_2_H_6_	0.010	20	
6FDA-6FpDA	CO_2_	0.045	20	[58]
	CH_4_	0.030	4.2	
	N_2_	−0.060	0.0	
	O_2_	0.015	0.0	
	C_2_H_4_	0.030	91	[59]
	C_2_H_6_	0.060	50	
	C_3_H_6_	0.070	66	
	C_3_H_8_	0.100	81	

**Table 5:** Penetrant infinite dilution mobility coefficient *L*_0_ and plasticization factor β_12_ at 35 °C for the various penetrants in the three glassy polymers inspected.

Polymer	Penetrant	$L_{12}^{0}$ (cm^2^/s)	β_12_	Ref. Exp.
PSf	CO_2_	9.0 × 10^−9^	17.5	[54]
	CH_4_	2.1 × 10^−9^	0.0	
	N_2_	6.4 × 10^−9^	0.0	
	Ar	1.1 × 10^−8^	0.0	
	He	3.4 × 10^−6^	0.0	
	H_2_	1.2 × 10^−6^	0.0	[55]
	O_2_	3.0 × 10^−8^	0.0	[56]
PPh	CO_2_	1.6 × 10^−8^	17.2	[57]
	CH_4_	4.7 × 10^−9^	5.0	
	N_2_	1.7 × 10^−8^	0.0	
	Ar	2.1 × 10^−8^	0.0	
	O_2_	4.9 × 10^−8^	0.0	
	C_2_H_6_	2.0 × 10^−10^	53.5	
6FDA-6FpDA	CO_2_	2.6 × 10^−8^	21.5	[58]
	CH_4_	5.5 × 10^−9^	6.0	
	N_2_	3.1 × 10^−8^	0.0	
	O_2_	8.3 × 10^−8^	3.0	
	C_2_H_4_	8.6 × 10^−10^	6.0	[59]
	C_2_H_6_	1.8 × 10^−10^	20.0	
	C_3_H_6_	4.8 × 10^−12^	137	
	C_3_H_8_	1.1 × 10^−12^	110	
